# Development and usability testing of a multifaceted intervention to reduce low-value injury care

**DOI:** 10.1186/s12913-024-12153-y

**Published:** 2025-01-07

**Authors:** Mélanie Bérubé, Alexandra Lapierre, Michael Sykes, Jeremy Grimshaw, Alexis F. Turgeon, François Lauzier, Monica Taljaard, Henry Thomas Stelfox, Holly Witteman, Simon Berthelot, Éric Mercier, Catherine Gonthier, Jérôme Paquet, Robert Fowler, Natalie Yanchar, Barbara Haas, Paule Lessard-Bonaventure, Patrick Archambault, Belinda Gabbe, Jason R. Guertin, Yougdong Ouyang, Lynne Moore, the Canadian Traumatic Brain Injury Research Consortium

**Affiliations:** 1 Population Health and Optimal Health Practices Research Unit, Centre de Recherche du CHU de Québec (Hôpital de L’Enfant-Jésus), Université Laval, 1401, 18e rue, Québec, Qc Canada; 2 Faculty of Nursing, Université Laval, 1050 Av. de La Médecine, Québec, Qc Canada; 3 Department of Social and Preventive Medicine, Université Laval, 1050 Av. de La Médecine, Québec, Qc Canada; 4 Department of Nursing, Midwifery, and Health, Northumbria University, Northumberland Road, Newcastle-upon-Tyne, UK; 5 Ottawa Hospital Research Institute, 725 Parkdale Ave, Ottawa (On), Canada; 6 Department of Anesthesiology and Critical Care Medicine, Université Laval, 1050 Av. de La Médecine, Québec, Qc Canada; 7 Faculty of Medicine & Dentistry, University of Alberta, 8440 112 ST NW, Edmonton (Ab), Canada; 8 Department of Family and Emergency Medicine, Université Laval, 1050 Av. de La Médecine, Québec, Qc Canada; 9 Institut national d’excellence en santé et en services sociaux, 2535 Bd Laurier, Québec, Qc Canada; 10 Sunnybrook Research Institute, 2075 Bayview Avenue, Toronto (On), Canada; 11 Department of Surgery, University of Calgary, 3280 Hospital Dr NW, Calgary (Ab), Canada; 12 Department of Surgery, University of Toronto, 149 College St, Toronto (On), Canada; 13 Department of Surgery, Division of Neurosurgery, Université Laval, 1050 Av. de La Médecine, Québec, Québec Canada; 14 School of Public Health and Preventive Medicine, Monash University, 553 St Kilda Rd, Melbourne, Victoria, VIC 3004 Australia

**Keywords:** Low-value practice, Trauma system, Intervention development, Multifaceted intervention

## Abstract

**Background:**

Multifaceted interventions that address barriers and facilitators have been shown to be most effective for increasing the adoption of high-value care, but there is a knowledge gap on this type of intervention for the de-implementation of low-value care. Trauma is a high-risk setting for low-value care, such as unnecessary diagnostic imaging and the use of specialized resources. The aim of our study was to develop and assess the usability of a multifaceted intervention to reduce low-value injury care.

**Methods:**

We used the Consolidated Framework for Implementation Research and the Expert Recommendations for Implementing Change tool as theoretical foundations to identify barriers and facilitators, and strategies for the reduction of low-value practices. We designed an initial prototype of the intervention using the items of the Template for Intervention Description and Replication. The prototype’s usability was iteratively tested through four focus groups and four think-aloud sessions with trauma decision-makers (*n* = 18) from seven Level I to Level III trauma centers. We conducted an inductive analysis of the audio-recorded sessions to identify usability issues and other barriers and facilitators to refine the intervention.

**Results:**

We identified barriers and facilitators related to individual characteristics, including knowledge and beliefs about low-value practices and the de-implementation process, such as the complexity of changing practices and difficulty accessing performance feedback. Accordingly, the following intervention strategies were selected: involving governing structures and leaders, distributing audit & feedback reports on performance, and providing educational materials, de-implementation support tools and educational/facilitation visits. A total of 61 issues were identified during the usability testing, of which eight were critical, 33 were moderately important, and 18 were minor. These issues led to numerous improvements, including the addition of information on the drivers and benefits of reducing low-value practices, changes in the definition of these practices, the addition of proposed strategies to facilitate de-implementation, and the tailoring of educational/facilitation visits.

**Conclusions:**

We designed and refined a multifaceted intervention to reduce low-value injury care using a process that increases the likelihood of its acceptability and sustainability. The next step will be to evaluate the effectiveness of implementing this intervention using a pragmatic cluster randomized controlled trial.

**Trial registration:**

This protocol has been registered on ClinicalTrials.gov (February 24th 2023, #NCT05744154, https://clinicaltrials.gov/ct2/show/NCT05744154).

**Supplementary Information:**

The online version contains supplementary material available at 10.1186/s12913-024-12153-y.

## Contributions to the literature

- Much research has been conducted to promote the use of high-value care in trauma, but few interventions have been proposed to reduce low-value care in this field, which is highly conducive to overuse and risk of harm to patients.
- Barriers and facilitators specific to low-value care and their underlying drivers may differ to those of high-value care. Therefore, interventions specifically designed for de-implementing low-value practices are required.
- We designed and refined a multifaceted intervention based on theorical foundations, evidence, and interested parties’ perspectives to reduce low-value injury care, which will advance knowledge in de-implementation science.

## Background

Suboptimal care is widespread, with around one third of patients receiving care for which harms and costs outweigh the benefits (i.e., low-value care) [1, 2]. This problem has considerable consequences for millions of people around the world, for healthcare systems, and for the environment [3]. As a result, the United Nations identified reducing low-value care through innovative solutions as an urgent task [4]. Traumatic injuries are the leading cause of death among young adults (< 45-year-olds) [5] and are associated with the highest number of productive years of life lost [6, 7]. Likewise, trauma represents one of the most resource-intensive diagnostic groups [5]. Therefore, interventions to improve adherence to best practices, including limiting low-value care, are crucial to the sustainability of services in the field of injury care.

Numerous efforts have been made over the last 30 years to address high-value care among healthcare professionals involved with trauma patients, which have been associated with positive outcomes such as decreased mortality and complications [8–11]. Nevertheless, given the urgency surrounding injury presentations and the need to make rapid decisions when multiple diagnostic and intervention options are possible, injury care is a high-risk setting for overuse [12]. In this context, evidence-based quality indicators targeting low-value injury practices were recently identified [13] and validated [14]. These low-value practices include diagnostic imaging and utilization of specialized resources (i.e., neurosurgical consultation in patients without significant cerebral injuries) when potentially not indicated, exposing patients to avoidable radiation exposure and/or unnecessary interventions on the basis of incidental findings, and increasing strain on healthcare resources [2, 15, 16].

Theory and evidence on the implementation of high-value care suggest that multifaceted interventions comprising at least two components [e.g., education and audit & feedback (A&F)] addressing determinants for success (i.e., barriers and facilitators) are most effective and cost-effective for improving clinicians’ practices [17–19]. However, barriers and facilitators for de-implementation may differ from those for implementation [19, 20]. Therefore, while there may be similarities in intervention strategies designed to implement and de-implement practices, there is a need for interventions that address the barriers and facilitators specific to low-value care. Accordingly, we designed a pragmatic cluster randomized controlled trial (RCT) to evaluate the effectiveness of a multifaceted intervention compared to simple A & F to reduce low-value acute injury care [12]. The trial is embedded into a national evaluation of trauma programs in the province of Québec, Canada, with local trauma committees required to analyze quality indicators and submit an action plan. The objectives of this study were to develop the multifaceted intervention and to assess its usability with trauma program leaders before proceeding to its evaluation.

## Methods

We developed the multifaceted intervention and refined it after usability testing in line with recommendations of the Medical Research Council guidelines for the Development of Complex Interventions to improve healthcare [21]. According to these recommendations, the development of interventions should be informed by theory, evidence and context while involving experts in the field through a partnership approach. We used the items of the Template for Intervention Description and Replication (TIDieR) [22] (i.e., why, what, who will provide, how, where, when and how much, how well, tailoring and modifications) to guide the design of the intervention. We also used the Consolidated Framework for Implementation Research (CFIR) [23] and the Expert Recommendations for Implementing Change (ERIC) [24] tool, as theoretical foundations for determining intervention strategies. This study is reported according to the Guidance for reporting intervention development studies in health research (GUIDED) (Supplemental digital file 1) [25]. The research project was approved by the CHU de Québec-Université Laval research ethics board (#113,664).

### Intervention development

To design the initial prototype of the multifaceted intervention, members of the research team with experience in intervention development and in quality improvement processes (M.B., M.S., L.M.) first listed the barriers and facilitators to reducing targeted behaviors—i.e., low-value injury practices selected based on expert consensus and a validation process (Table 1) [13, 14]. These barriers and facilitators, were either identified by experts (i.e., surgeons, emergency physicians, intensivists, nurses, trauma program managers) during our international consensus study aimed at prioritizing practices that should be targeted for de-implementation [13] or in a recent systematic review [26]. Then, we linked barriers and facilitators to the CFIR constructs embedded in the five domains of the framework [23]: 1) intervention characteristics, 2) outer setting, 3) inner setting, 4) characteristics of individuals, and 5) implementation process. This exercise enabled us to identify the relevant CFIR constructs and match them with the ERIC tool [24]. This tool contains 73 strategies grouped under nine categories: 1) engage consumers, 2) use evaluative & iterative strategies, 3) change infrastructure, 4) adapt & tailor to the context, 5) develop stakeholders interrelationships, 6) utilize financial strategies, 7) support clinicians, 8) provide interactive assistance, and 9) train & educate stakeholders. [24] To facilitate the process of identifying intervention strategies, we used the CFIR-ERIC Matching tool (https://cfirguide.org/choosing-strategies/), which allowed us to generate a list of the most promising strategies based on research data and consensus of experts in implementation science [24].

**Table 1:** Low-value practices targeted by the multifaceted intervention

Category of practices potentially not indicated	Low-value practice
Initial diagnostic imaging	• Head CT in low-risk patients^a^ • Cervical spine CT in low-risk patients^a^ • Whole body CT in patients with minor injuries and single-system injury
Specialist consultation	• Neurosurgical consultation in patients without significant cerebral injuries
Repeat imaging for transfers	• Pre-transfer imaging in patients with a clear indication for transfer • Repeat post-transfer CT in patients with no disease progression and no additional details needed

^a^Low risk patients according to a validated clinical decision rule (i.e. the Canadian Computed Tomography Head Injury Rule and the Canadian Cervical Spine Rule): minor injury, low-velocity injury mechanism, Glasgow Coma Score of 15 and hemodynamically stable, not receiving anticoagulant, no open or depressed skull fracture, no evidence of skull base fracture, no spinal pathology

*CT* Computerized tomography

The identification of strategies, according to the CFIR and ERIC, enabled us to determine the goal of the elements essential to the intervention (i.e., the *why* item of the TIDieR) [22], the potential content of the intervention and the materials and procedures (i.e., the *what* and *how* items of the TIDieR) [22]. These strategies and the context in which the intervention was planned to be implemented (i.e., provincial quality assurance program) were then considered to establish its other features: how the activities would be carried out (*by whom and where*), their timing (*when*) throughout the performance evaluation cycle according to the needs of trauma teams (tailoring), their duration (*how much*) and the assessment of their fidelity (*how well*) [22].

### Usability testing

We iteratively conducted four focus groups [27, 28] and four think-aloud sessions [29–31] from May 2022 to September 2023, to test the usability of the intervention prototype and refine it until no moderate to critical issues likely to hamper the intervention’s implementation were identified [32]. In accordance with recommendations, focus groups included three to five participants [27, 33], and the think-aloud sessions, one to two participants [34, 35]. Participants in the focus group and think-aloud sessions included at least one trauma program leader (i.e., medical director, trauma program manager, quality improvement service manager) concerned by the targeted practices from seven Level I to Level III trauma centers, representing various Québec regions, that were to be randomized to the pragmatic cluster RCT intervention group. These leaders, whose contact details were obtained from the organization overseeing the performance evaluation of trauma centers in Québec, the *Institut national d'excellence en santé et en services sociaux* (INESSS), were contacted by e-mail to determine their interest in participating in the study. In some cases, department heads (e.g., radiology, emergency department) were invited by trauma program leaders to join the focus groups or the think-aloud sessions. A scientific coordinator and a scientific professional from INESSS were also consulted for feedback on the intervention prototype at the end of the process through a think aloud session.

Focus groups and think-aloud sessions were conducted virtually according to recognized standards [36] by researchers (M.B., L.M.) trained in moderating group discussions and conducting usability testing. Intervention-related material was sent by e-mail in PDF format to participants the week prior to focus groups and think-aloud sessions and was projected on screen during the meetings. To conduct the focus groups, we used a semi-structured questionnaire [27] (Supplemental digital file 2) to gather feedback on the intervention content, materials and procedures, generate ideas for potential modifications, and identify other potential barriers and facilitators using the CFIR [23]. For the think-aloud sessions, we used a protocol (Supplemental digital file 3) that led participants to express their reactions and understanding while reviewing the intervention content and materials [30] These sessions also aimed to identify other barriers and facilitators for implementing the intervention.

**Fig. 1:**
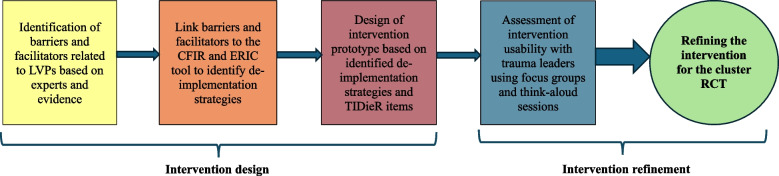
Process used for designing and refining the intervention. CFIR: Consolidated Framework for Implementation Research; ERIC: Experts Recommendations for Implementing Change; LVPs: Low-value practices; RCT: Randomized controlled trial; TIDieR: Template for Intervention Description and Replication

After each usability assessment, we conducted inductive content analysis [27, 37] from the audio-recorded focus groups and think-aloud sessions using verbatim transcriptions to proceed with the required modifications. Codes were generated to classify usability issues according to severity metrics commonly used in usability assessments [31, 38]: 1) minor issues referring to typos, legibility, or esthetic preference, 2) moderate issues, such as difficulty understanding or using the intervention materials as designed, and 3) critical issues, such as technical problems that prevent participants from using the intervention materials in a way that fulfils its purpose, i.e. to establish an action plan to de-implement low-value practices. Coding and issues identification were carried out independently in duplicate by a senior research team member with expertise in usability testing (M.B.) and a post-doctoral fellow (A.L.) [31]. Figure 1 illustrates the process of designing and refining the intervention.

## Results

### Intervention development

The way in which the barriers and facilitators identified in our previous consensus study [13] and those described in a recent systematic review [26] relate to the CFIR domains and constructs is presented in Table 2. Barriers and facilitators were similar across low-value practices and concerned the inner setting, including tension for change, compatibility (i.e., how the intervention aligns with individuals’ own norms, values, and perceived risks and needs, and how it fits with existing workflows and systems), ease of access to digestible information, and knowledge about the intervention and how to incorporate it into work tasks. Barriers linked to the individual characteristics construct were also frequently identified, such as the perceived advantage of implementing the intervention, knowledge and beliefs about low-value practices, and perceived self-efficacy for de-implementing them. The same was true for barriers and facilitators related to the characteristics of the intervention and the de-implementation process, including the perceived benefits of the intervention, the complexity of changing practices and the difficulty of accessing performance feedback, as well as the need to involve leaders and be supported in planning the de-implementation. Accordingly, the following intervention strategies, focusing on individuals and system changes were selected: 1) involve governing structures including the Trauma Center Chief Executive Officer, 2) audit and provide feedback on targeted low-value practices through a recognized national authority for the evaluation of practice standards, 3) provide educational material and de-implementation support tools (i.e., summary of findings from clinical trials on low-value practices, decision support tools, share-decision making tools, guideline recommendations), 4) offer virtual education meetings, 5) provide virtual facilitation visits, and 6) conduct local needs assessments regarding the intervention (e.g., trauma program managers, trauma program medical directors, head of targeted services).

**Table 2:** Matching of barriers and facilitators with implementation strategies according to the CFIR-ERIC tool

Barriers and facilitators identified in previous consensus study and the literature	The why of the intervention		
	**CFIR domains**	**Type of barriers and facilitators according to the CFIR**	**Implementation strategy according to the CFIR-ERIC matching tool**
Beliefs and opinions of providers on low-value practices	• Intervention characteristics • Inner setting • Characteristics of individuals	• Relative advantage • Culture • Knowledge and beliefs about the intervention	• Identify and prepare champion • Conduct educational meeting
Fear of medical error by de-implementing low-value practice	• Intervention characteristics • Inner setting	• Relative advantage • Tension for change • Compatibility	• Identify and prepare champion • Conduct local consensus discussion • Promote adaptability
Defensive attitude (better to provide the low-value practice in the presence of doubt)	• Intervention characteristics • Inner setting	• Relative advantage • Culture • Tension for change • Compatibility	• Identify and prepare champion • Conduct local consensus discussion
Desire to meet patients’ expectations	• Outer setting • Implementation process	• Patient needs and resources • Engaging patients/customers	• Obtain and use patients/consumers and family feedback • Involve patients/consumers and family members • Conduct local needs assessment • Provide shared decision-making tools
Motivation and commitment to restrict unnecessary care	• Inner setting • Characteristics of individuals	• Goals and feedback • Self-efficacy • Individual stage of change	• Audit & provide feedback • Develop a formal implementation blueprint • Provide ongoing consultation • Conduct ongoing training • Make training dynamic
Provider knowledge about low-value practices	• Inner setting • Characteristics of individuals	• Access to knowledge and information • Knowledge and belief about the intervention	• Conduct educational meeting • Develop and distribute educational material
Awareness of an agreement with guidelines	• Inner setting • Characteristics of individuals	• Access to knowledge and information • Knowledge and belief about the intervention	• Conduct educational meeting • Develop and distribute educational material • Provide clinical decision rules
Sense of ownership and participation in the project	• Inner setting • Implementation process	• Learning climate • Leadership engagement • Opinion leaders • Formally appointed internal implementation leaders • Champions	• Facilitation • Involve executive boards • Identify and prepare champion • Inform local opinion leader
Lack of database to monitor the quality of care and progress of de-implementation	• Inner setting • Implementation process	• Goals and feedback • Reflecting and evaluating	• Audit & provide feedback • Develop and implement tools for quality monitoring
Having the right information from databases	• Inner setting • Implementation process	• Goals and feedback • Reflecting and evaluating	• Audit & provide feedback • Develop and implement tools for quality monitoring
Lack of time	• Intervention characteristics • Inner setting	• Complexity • Available resources	• Develop a formal implementation blueprint • Promote adaptability • Access new funding
Lack of tools to facilitate change in practice	• Intervention characteristics • Inner setting • Characteristics of individuals • Implementation process	• Complexity • Available resources • Self-efficacy • Planning	• Develop a formal implementation blueprint • Promote adaptability • Access new funding • Provide ongoing consultation • Conduct ongoing training • Make training dynamic • Conduct local needs assessment
Change required in the existing workflow or referral patterns	• Intervention characteristics • Inner setting • Characteristics of individuals • Implementation process	• Complexity • Compatibility • Learning climate • Self-efficacy • Planning	• Develop a formal implementation blueprint • Promote adaptability • Facilitation • Provide ongoing consultation • Conduct ongoing training • Make training dynamic • Conduct local needs assessment • Assess and redesign workflow
Economic political context financial incentives to do less	• Outer setting	• External policy & incentives	• Involve executive board • Alter incentive/allowance structure

*CFIR* Consolidated Framework for Implementation Research; ERIC: Experts Recommendations for Implementing Change

**Fig. 2:**
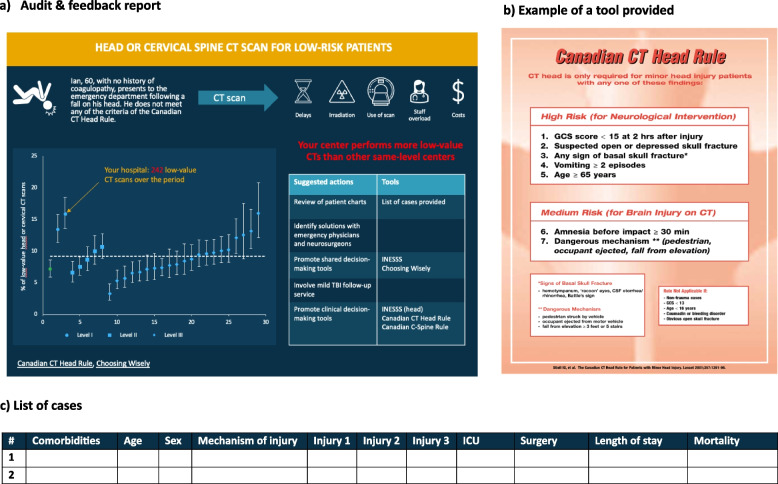
Example of initial intervention prototype for head or cervical spine CT in low-risk patients

Each of these strategies, along with a description of who is responsible for applying them, when they are to be applied, how long they are to last, and how the fidelity of their use will be assessed, are presented in the intervention prototype in Table 3. Also, the initial prototype of the intervention for one of the low-value practices (i.e., initial head/spine CT), with associated materials (A & F report, educational content) is shown in Fig. 2.

**Table 3:** Intervention prototype as per the TIDieR items

Implementation strategies (what and how)	By whom (who) to which interested parties in level I-III trauma centers in Quebec (where)	Timing (when), duration (how much) and tailoring	Fidelity assessment (how well)
Involve governing structures: • Involve the organization responsible for developing accreditation standards (INESSS) in the production and distribution of audit & feedback (A&F) reports • Send A&F report to trauma center Chief Executive Officers (CEOs) through a secured national web platform • Send A&F report to trauma medical director and trauma program manager through through a secured national web platform	By: Members of the research team^a^, INESSS To: CEOs, trauma medical director, trauma program manager	During the production of the A&F (INESSS) Beginning of each evaluation cycle	• Report consultation by CEO, medical trauma directors and trauma program coordinators (verified with Google Data Analytics)
• Audit & provide feedback through a secured national web platform: • Performance compared to peers (simple A&F)^b^ • Performance over time^c^ • Summary message indicating if action is required	By: Members of the research team^a^, INESSS To: CEOs, trauma medical director, trauma program manager	Beginning of each evaluation cycle	• Report consultation by CEO, medical trauma directors and trauma program coordinators • Questions from CEO, medical trauma directors and trauma program coordinators regarding the report content
• Provide educational material and de-implementation support tools in a written document through a secured national web platform**:** • Preliminary list of suggested actions based on matching of barriers/facilitators to ERIC strategies (e.g., inform local opinion leaders, conduct local needs assessment through chart reviews) • Clinical vignettes on LVPs • Consequences of LVPs • Links to supporting materials (clinical practice guidelines, clinical decision rules, clinician-patient/family shared decision-making tools) • Patient chart revision tool	By: Members of the research team^a^, INESSS To: Members of local trauma committees	Beginning of the first evaluation cycle	• Use of the chart revision tool for producing the action plan • Uploading of the educational materials by medical trauma directors and trauma program coordinators (verified with Google Data Analytics) • Questions from medical trauma directors and trauma program coordinators regarding the educational material
Virtual educational meeting: • Presentation on quality indicators, their rationale and how to interpret results • Training on how to evaluate barriers and facilitators and select solutions with members of local trauma committee using the CFIR-ERIC matching tool	By: Members of the research team^a^ To: Trauma medical director, trauma program manager and data information specialist	Beginning of the first evaluation cycle, within 2 to 6 weeks following the transmission of the A&F report^b^ Duration: 60–90 min	• Delivery of educational components as planned • Examination of action plan to determine whether they address the feedback provided and describe the strategies suggested to facilitate the de-implementation of LVPs based on a local assessment of barriers and facilitators
Virtual facilitation visits-Ongoing consultation: • Interpretation of feedback • Identification of barriers and facilitators to establish their action plan • Provide support on how to tailor their action plan to meet local needs while addressing the most important issues (promote adaptability)	By: Members of the research team^a^ To: Trauma medical director, trauma program manager	2 and 4 months^d^ after transmission of the A&F report or at other more appropriate times depending on the needs of trauma teams^b^ Duration: 30–60 min	• Delivery of facilitation visit components as planned • Examination of action plan
Conduct local needs assessment regarding the intervention and prepare champions: Participation of local trauma committee members and other key end users in the refinement of the intervention prototype (virtual focus groups and think-aloud sessions)	By: Members of the research team^a^ To: Members of local trauma committees: Trauma medical directors, trauma program manager, heads of emergency department and radiology, representative from provincial quality improvement program	Before the implementation of the intervention and after its implementation during the process evaluation Duration: Four 60-min focus groups and four 60-min think-aloud sessions for usability testing	NA

^a^The research team includes a trauma epidemiologist, a doctoral trained nurse practitioner specialized in injury care, who has been a trauma program manager for several years, and a post-doctoral fellow specialized in emergency care for the trauma population

^b^Simple A&F (control intervention)

^c^The same components could be applied to both evaluation cycles depending on staff turnover and according to local needs

^d^The 2- and 4-month timeline was established based on our previous experience with the implementation of high-value care based on audit & feedback report. A&F: Audit & Feedback; CEO: Chief executive officer; INESSS: Institut National d’Excellence en Santé et en Services Sociaux; LVP: Low-value practice; NA: not applicable

### Usability testing

Table 4 displays the characteristics of trauma centers and participants involved in the usability testing. All the trauma teams approached agreed to take part in the study. Most usability assessments took place in Level I and Level II trauma centers in urban areas (71%). In addition, most of the participants were trauma medical directors and trauma program managers (66%) with five years or less experience in a clinical-administrative trauma role (83%).

**Table 4:** Characteristics of participants and trauma centers for the usability testing phase

**Characteristics**	**n (%)**
Role (*N* = 18) Trauma medical director (i.e., surgeons, emergency physicians) Trauma program manager Quality improvement manager Head of services (i.e., radiology, emergency department)	6 (33) 6 (33) 3 (17) 3 (17)
Gender (*N* = 18) Male Female	8 (44) 10 (56)
Year(s) of experience in a clinical-administrative trauma role (N = 18) 1–2 years 3–5 years > 5 years	8 (44) 7 (39) 3 (17)
Level of trauma center (*N* = 7) Level I Level II Level III	3 (42) 2 (29) 2 (29)
Geography of trauma center (*N* = 7) Urban Rural	5 (71) 2 (29)

Table 5 describes the moderate and critical issues identified during the refinement process. Although our initial aim was to iteratively conduct the focus group and think-aloud sessions, we carried out more focus groups at the outset to obtain further information on the modifications to be made to the intervention. The focus groups and think-aloud sessions lasted 60 (± 10) minutes on average. We identified a total of 61 issues: 8 critical, 33 moderate, and 18 minor. Critical issues included situations such as incomplete information about the drivers of each low-value practice, lack of information about the benefits of low-value practice reduction and the inclusion of patients potentially not targeted by low-value practices in the lists of medical records to be reviewed. Moderate issues mainly concerned the need to clarify certain definitions and drivers of low-value practices, the addition of details to the list of medical records to be reviewed to better capture patients’ medical condition (e.g., imaging, referral hospitals), and suggestions for additional strategies to facilitate the de-implementation of practices. Minor problems were related to wording, quality of images, background color, and the inclusion of hyperlinks to access scientific articles and guidelines. As a result, we made 60 modifications to the intervention to resolve the issues identified: 16 for all low-value practices, 13 for repeat CTs, 10 for initial head/spine CT, five for the initial whole-body CT, two for neurosurgical consultation, four for the background information provided on the introductory page, and 10 linked to formatting elements.

**Table 5:** Moderate and severe issues identified during the intervention refinement process

Data collection	Issues (n)	Low-value practice	Issue	Severity
**Focus group #1 (Level I trauma center)**	4	All LVPs	There is no list of medical records to be reviewed with appropriate care (patients who did not receive the LVP)	Critical
		Repeat CTs	Body region information is missing from the patient list for repeat CTs	Moderate
			The fact that the image is transferred but no report is sent from the referring center is a missing cause of the repeat CTs	Moderate
		Neurosurgical consultation	Institutional protocols could be a relevant strategy to add to facilitate the de-implementation of neurosurgical consultation	Moderate
**Focus group #2 (Level II regional trauma center)**	7	All LVPs	The definition of each appropriate care practice is missing	Critical
			The determinants of each LVP are missing	Critical
			Information on the benefits of LVP reduction is missing from all reports	Critical
			Reports lack information about the risks of using LVPs	Critical
			The title of the table listing patients with appropriate care is confusing	Moderate
		Initial head/spine CT	The list of medical records to be reviewed includes patients with moderate to severe facial trauma	Critical
		Background information	The description of support sessions does not mention the possibility of additional sessions. These could be relevant	Moderate
**Focus group #3 (Level II trauma center)**	5	All LVPs	Some clinical practice tools are available in English only	Moderate
			The meaning of the acronyms ISS and AIS is unknown to some participants	Moderate
			Support in presenting their A&F report to trauma leaders could be a strategy to add to facilitate the de-implementation of LVPs	Moderate
		Repeat CTs	Unstable patients should not be included in the list of medical records of pre-transfer CTs	Critical
			The patient's transfer status is missing from the LVP repeat CT list	Moderate
**Think aloud #1 (Level I trauma center)**	5	Repeat CTs	The strategy "harmonizing imaging protocols across centers" is missing	Moderate
			Lack of relevant or incomplete imaging is a missing reason for repeat CTs prior to transfer	Moderate
		Initial head/spine CT	The benefit of reducing clinically non-significant incidental findings is unclear	Moderate
		Initial whole-body CT	Information about other scans performed is missing	Moderate
		Background information	The "30% of the health budget" in the background information requires clarification	Moderate
**Focus group #4 (Level II regional trauma center)**	11	All LVPs	Journal club is a relevant strategy that could support knowledge translation regarding all LVPs	Moderate
		Repeat CTs	Fear of missing an injury is one cause of repeated CTs that could be added	Moderate
			“Availability of out-of-hours radiology transcription” should be considered as barrier to avoid repeating CTs	Moderate
			“Radiologist billing” should be considered as barrier to avoid repeating CTs	Moderate
		Initial head/spine CT	Inclusion and exclusion criteria for the use of clinical decision rules are lacking	Critical
			It should be mentioned that only admitted patients (not emergency room patients) are included in the data	Moderate
		Initial whole-body CT	"No increase in length of stay" should be added to the evidence supporting the deimplementation of initial whole-body CT in low-risk patients	Moderate
			The Western Trauma Association guidelines that promote whole-body CTs should be considered as a barrier for the de-implementation of this LVP	Moderate
			Clarification is needed on the definition of a whole-body CT	Moderate
		Neurosurgical consultation	Sometimes, neurosurgical consultation is used to ensure follow-up by only one physician, thereby improving continuity of care	Moderate
		Background information	The reality in non-academic centers differs from that of major trauma centers due to a lack of resources	Moderate
**Think aloud #2 (Level II trauma center)**	5	All LVPs	There is a lack of information about the mechanism of trauma in LVP definitions	Moderate
			The year and admission date are missing from the list of medical records to be reviewed	Moderate
		Repeat CTs	Destination hospitals for pre-transfer LVPs are missing from the list of medical records to be reviewed	Moderate
		Initial head/spine CT	The fact that reducing imaging reduces patient anxiety is not entirely true and should be removed	Moderate
		Initial whole-body CT	Recommendations on which patients should have a whole-body CT are lacking	Moderate
**Think aloud #3 (Level I trauma center)**	5	All LVPs	Internal quality improvement process is a relevant strategy for all LVPs and is missing	Moderate
		Repeat CTs	The referral center is missing from the list of patients admitted to a Level I center	Moderate
			Need to add “if images are adequate” to the risk section	Minor
		Initial head/spine CT	The fact that there are TBI follow-up clinics is not a strong enough argument not to ask for a head CT, as these follow-ups as conducted in the post-acute phase	Moderate
		Initial CTs and repeat CTs	The ecological impact is an aspect that has been raised, and that does not appear in the benefits	Moderate
**Think-aloud #4 (INESSS)**	10^a^	General comments	Only minor issues: Wording, colors, image quality, actionable hyperlinks	

^a^A total of 18 minor issues were identified during the focus groups and think-aloud sessions including 10 during the think-aloud session #4

*AIS* Abbreviated injury scale, *CT* Computed tomography, *ISS* Injury Severity Score, LVP

**Fig. 3:**
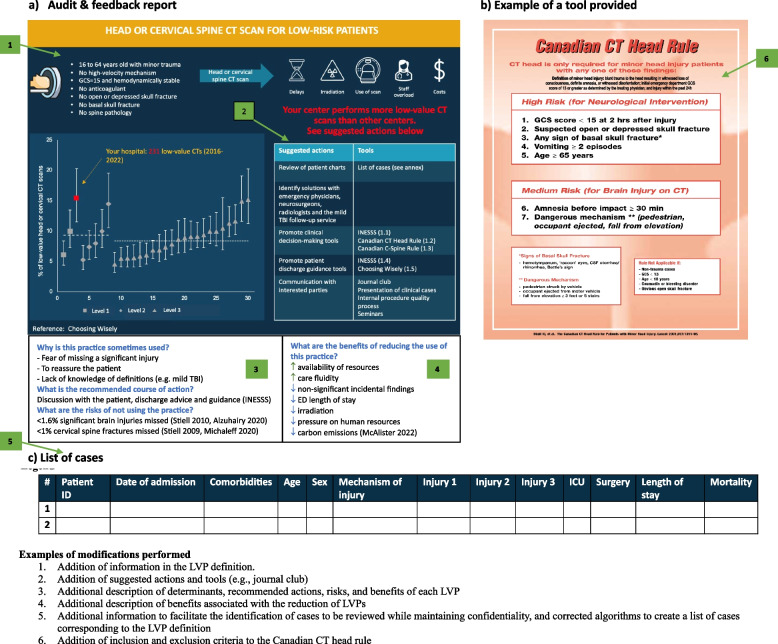
Example of refined intervention prototype for head or cervical spine CT in low-risk patients

We did not identify any new barriers or facilitators that contributed to the design of the multifaceted intervention through the usability testing process. Nevertheless, the importance of taking the following barriers/facilitators into account was reinforced: beliefs and opinions of providers on low-value practices; fear of medical error caused by de-implementing these practices; and lack of tools to facilitate changes. Accordingly, additional information on the benefits (e.g., improved care fluidity, decreased pressure on human and material resources, fewer clinically non-significant incidental findings, lower carbon emissions) of reducing low-value practices and evidence (e.g., no increase in mortality or morbidity) showing minimal risk associated with reductions were included. Further strategies to facilitate the de-implementation of low-value practices were also added to the preliminary list (e.g., developing institutional protocols, harmonizing imaging protocols across centers, journal clubs) and additional educational and support visits will be offered to trauma center teams as needed. Figure 3 shows an example of the modifications made as a result of usability testing for the initial head/spine CT low-value practice.

## Discussion

In this study, we designed and refined a multifaceted intervention to facilitate the de-implementation of low-value practices in injury care. The CFIR [23] and the ERIC [24] tool enabled us to design an intervention based on barriers and facilitators to the reduction of targeted practices identified by trauma interested parties and according to evidence. Barriers and facilitators mainly concerned healthcare professionals' beliefs and perceived benefits, as well as the self-efficacy and the complexity of practice change in organizations. As a result, an intervention comprising numerous strategies to de-implement low-value practices was designed. These strategies focus on individuals and system changes and include distributing annual A & F reports on performance through a recognized national authority for the evaluation of practice standards, involving trauma center leaders, and providing educational materials and de-implementation support tools and educational/facilitation visits.

Through usability testing among interested parties from trauma centers of different designation levels and representatives from a national body overseeing the performance evaluation of trauma centers, we identified critical, moderate and minor issues that led to multiple improvements to the intervention. Improvements included the addition of information on drivers and benefits of reducing targeted low-value practices, changes in the definition of low-value practices, the addition of proposed strategies to facilitate de-implementation, and the tailoring of the educational/facilitation visits to the needs of trauma teams.

Obtaining interested parties’ input on barriers and facilitators to the development of interventions aimed at improving clinical practices has been identified as a crucial step in increasing their penetration into the healthcare system, their perceived relevance, and their sustainability [23, 39]. This approach is also in line with the Choosing Wisely De-Implementation Framework (CWDIF), that was designed to promote the implementation of evidence-based strategies to effectively reduce low-value care [40]. Barriers and facilitators are considered as determinants of healthcare professionals’ behaviors [41]. Several of these determinants were identified as hindering or enabling the adoption of recommended care and the reduction of low-value care. As shown in our study, the barriers and facilitators most often identified as being involved in improving practices are related to healthcare professionals and the organizational context [26, 42–44]. Healthcare professionals’ beliefs, attitudes, knowledge, understanding, skills and confidence have been recognized as important determinants to consider in implementation and de-implementations processes [26, 42–44]. The same applies to organizational leadership and support for practice improvement, the efforts required to change practices, and the availability of data to monitor quality of care and progress [26, 42, 43].

Despite similarities between the categories of barriers and facilitators identified for high- and low-value practices, the underlying drivers associated with the latter may make de-implementation different to implementation [26]. These drivers include healthcare professionals’ fear of the consequences of withholding a test or treatment, their desire to meet patients' expectations and the time needed to explain the non-necessity of a treatment, their willingness to adopt new treatments rather than abandon existing ones, and financial incentive [26, 45]. Hence, interventions that address barriers and facilitators need to be designed diligently to facilitate the reduction of low-value care [45, 46].

A variety of strategies have been incorporated into interventions aimed at reducing low-value practices, such as those selected in our study [45–47]. A recent knowledge synthesis showed that the following ERIC strategies are the most frequently used in de-implementation interventions: training and education (i.e., educational materials, making training dynamic by promoting the active participation of interest parties, organizing educational meetings), the use of evaluative and iterative strategies (i.e., A & F, implementing a quality monitoring system) and support for clinicians (i.e., using a clinical decision support tool) [47]. Similarly, strategies related to behavior substitution (i.e., replacing low-value practices by a desired practice, for example by using share-decision tools to better communicate with patients) and restructuring the social environment (e.g., requiring discussion of care with other colleagues or obtaining authority approval to use a potentially low-value practice) were found to be more frequently used in de-implementation interventions than in implementation interventions [48].

Regardless of the number of strategies that make up an intervention, those that contain educational materials or involve the use of A&F were shown to be the most effective in reducing low-value practices [45, 46]. Also, the combination of educational materials and meetings, A&F, patient-directed strategies (e.g., educational material, share-decision making tools), and organizational strategies (e.g., tools for supporting decision-making, changing test ordering procedures and forms for de-listing practices) were shown to enhance the sustainability of the effect [46]. However, in order to overcome the powerful underlying drivers associated with low-value care, it was recognized that problem analysis and context assessment were fundamental to tailoring de-implementation strategies [45, 46]. This exercise was made possible in our study through usability testing, which enabled us to refine the intervention with interested parties to strengthen the ability of trauma teams to de-implement the targeted practices.

Previous systematic reviews have showed favorable outcomes associated with de-implementation interventions [45, 46, 49–51] including a recent review of RCTs that found a significant median relative reduction of 17% in low-value care [46]. However, a greater number of interventions tailored to contextual factors, as in our study, were recognized as a potential strategy for further increasing their impact on low-value care [45, 46]. In addition, most of the de-implementation interventions whose effectiveness has been evaluated to date have focused on medication in a primary care setting [46], and there is still limited evidence on emergency care, including trauma-specific de-implementation interventions. The field of trauma differs from many other healthcare areas due to the complexity of clinical practice resulting from the speed of decision-making, heterogeneous populations, the multiplicity of specialists involved, the wide range of diagnostic and therapeutic options, and the need to transfer many patients to specialized centers for definitive care [12]. The potential benefits of de-implementation interventions adapted to this context therefore need to be further investigated. Hence, the next step of this research project will be to evaluate the effectiveness of the developed multifaceted intervention on the reduction of targeted low-value practices [12].

### Study strengths and limitations

This study proposes an intervention aimed at reducing low-value practices in acute injury care, that is based on a rigorous process of design and refinement. The description of the process and the results obtained will help to advance the science of de-implementation in the field of emergency care and in other healthcare areas. Similarly, prior identification of low-value practices according to a thorough review of the evidence [52–55] and expert consensus [13], comprehensive analysis of barriers and facilitators to determine intervention strategies, consultation with interested parties to refine these strategies, and the embedment of the process within a system-based initiative, increase the likelihood of having developed an acceptable, effective and sustainable intervention [23, 39, 40, 56].

This study also has limitations. First, the identification of barriers and facilitators that contributed to the selection of the intervention strategies was not carried out through direct clinical observations in each of the trauma centers involved in the provincial performance evaluation process. However, experts working in several centers across Canada and internationally were consulted and a systematic review of the most common barriers and facilitators in de-implementation approaches was used, increasing the probability that we targeted the most important ones. Second, intervention refinement was conducted only with trauma decision-makers, the majority of whom had less than 5 years’ experience in this role, and in a small number of rural trauma centers (Level III). Trauma medical directors and trauma program managers are responsible for ensuring the quality of patient care, and thus for analyzing performance data and working with the multidisciplinary team to develop improvement plans [57]. They were therefore the key interested parties to involve in refining the intervention to promote appropriate initiation of low-value practice de-implementation. The processes surrounding de-implementation will be evaluated with healthcare professionals concerned by the low-value practices as part of our clinical trial, enabling us to gather their feedback and continue to improve the intervention for future performance evaluation cycles. The same is true for the planned educational and facilitation sessions, which will not only inform us about further refinements required to better meet the needs of trauma centers according to their level of designation, but will also provide support for less experienced decision-makers.

## Conclusion

This study led to the design and refinement of a multifaceted intervention to facilitate the reduction of low-value injury care, based on theoretical frameworks, evidence, and a consultation with interested parties. Barriers and facilitators to the de-implementation of targeted practices, mainly related to healthcare professionals and organizational context, were identified. These led us to design an intervention comprising multiple components, which were then refined by addressing important usability issues. The next steps will be to evaluate the effectiveness of the intervention through a pragmatic cluster-RCT, while assessing de-implementation processes to better understand the contextual factors that hinder or facilitate the reduction of low-value injury care.

## Supplementary Information

Supplementary Material 1.

Supplementary Material 2.

Supplementary Material 3.

## Data Availability

All data generated or analysed during this study are included in this published article.

## Abbreviations

A&F: Audit & feedback
CFIR: Consolidated Framework for Implementation Research
CT: Computed tomography
CWDIF: Choosing Wisely De-Implementation Framework
ERIC: Expert Recommendations for Implementing Change
GUIDED: Guidance for reporting intervention studies in health research
INESS: Institut national d’excellence en santé et en services sociaux
LVP: Low-value practice
RCT: Randomized controlled trial
TIDieR: Template for Intervention Description and Replication
