# Supplementary material for: Development and usability testing of a multifaceted intervention to reduce low-value injury care
Source: BMC Health Serv Res. 2025 Jan 7;25:37. doi: 10.1186/s12913-024-12153-y (PMC11706146; doi:10.1186/s12913-024-12153-y)
Supplement: Supplementary file 2 — Supplementary Material 2. [file 12913_2024_12153_MOESM2_ESM.docx]

**Supplemental digital file 2. Questions for the semi-structured interviews (focus groups)**

1. What do you think in general of the intervention?
2. What types of changes or modifications do you think we should make to the intervention to facilitate its integration into your setting?
3. Are there any components of the intervention that should not be changed? If so, which ones?
4. What is your perception of the quality of support materials and the presentation of the intervention to facilitate its implementation? What types of information and materials regarding the intervention could be provided for individuals at your facility?
5. What do you think are the potential benefits of the intervention for the patients provided for by your organization? For instance, do you believe that the implementation of the intervention will result in better access to services, a reduction in waiting time, a decrease in adverse events?
6. What are your thoughts on current practices related to the intervention? How will the intervention address current gaps in practice?
7. What kinds of changes will be needed to implement the intervention (Changes in scope of practice? Changes in official policies? Changes in information systems or electronic record systems? Others?). Can you describe the processes that will be required to make these changes?
8. How do you think your organization’s culture (general beliefs, values that people hold about wasting resources and costs) will affect the implementation of the intervention?
9. How confident are you that you will be able to successfully implement the intervention and what gives you that level of confidence (or lack of)?
