# Supplementary material for: Development and usability testing of a multifaceted intervention to reduce low-value injury care
Source: BMC Health Serv Res. 2025 Jan 7;25:37. doi: 10.1186/s12913-024-12153-y (PMC11706146; doi:10.1186/s12913-024-12153-y)
Supplement: Supplementary file 3 — Supplementary Material 3. [file 12913_2024_12153_MOESM3_ESM.docx]

**Supplemental digital file 3. Think-aloud protocol**

- I am going to ask you to think aloud when using the intervention tools. Let me explain what I mean by "think aloud." This means that I would like you to tell me everything you are thinking about as you use each intervention tool. You will do this one tool at a time.
- When I say "tell me everything," I really mean every thought you have from the moment you interact with the tool until the end of its use. Don't worry about planning how to say things or clarifying your thoughts. What I really want is to hear your thoughts all the time while you are using the tools. You may need time to quietly think about something - if so, that's okay, but let me know what you thought as soon as possible after you're done.
- I realize it can feel awkward to think aloud but try to imagine you are alone in the room. If you become silent for too long, I will say “keep talking” to remind you to think aloud. Please note, this research is highly exploratory. My intention is not to evaluate your thinking or explanations while you speak. The purpose of the study is to learn about the thoughts as you—and other people—have while using the intervention tools.
- We are going to do a warm-up activity to help you feel comfortable with thinking aloud. What questions do you have before we get started with some practice?
  - What is the fifth letter before “M” in the alphabet? Now, please tell me everything you think about as you answer this question.
  - Name five animals that live at the zoo. Now, please tell me everything you think about in the process of finding these five animals?

Adapted from:

Johnson et al. (2022). Using the think aloud protocol in health professions education; an interview method for exploring thought process: AMEE Guide No.151. Medical Teacher, doi: 10.1080/0142159X.2022.2155123

Wolcott et al. (2021). Using cognitive interviews and think-aloud protocols to understand thought processes. Currents in Pharmacy Teaching and Learning, 13:181-188
